# Supplementary material for: A compact tunable polarized X-ray source based on laser-plasma helical undulators
Source: Sci Rep. 2016 Jul 5;6:29101. doi: 10.1038/srep29101 (PMC4932604; doi:10.1038/srep29101)
Supplement: Supplementary Information [file srep29101-s1.pdf]

Supplementary materials of "A compact tunable polarized X-ray source based on laser-plasma helical undulators"

Authors:

J. Luo[1,2], M. Chen[1,2*], M. Zeng[1,2], J. Vieira[3], L.L. Yu[1,2], S.M. Weng[1,2], L.O. Silva[3], D.A. Jaroszynski[4], Z.M. Sheng[1,2,4†], and J. Zhang[1,2]

Affiliations:

[1]Key Laboratory for Laser Plasmas (Ministry of Education) and Department of Physics and Astronomy, Shanghai Jiao Tong University, Shanghai, 200240, China.
[2]Collaborative Innovation Center of IFSA (CICIFSA), Shanghai Jiao Tong University, Shanghai 200240, China.
[3]GoLP/Instituto de Plasmas e Fusao Nuclear, Instituto Superior Tecnico, Universidade de Lisboa, Lisbon 1049-001, Portugal.
[4]SUPA, Department of Physics, University of Strathclyde, Glasgow G4 0NG, UK.
*e-mail: minchen@sjtu.edu.cn
†e-mail: z.sheng@strath.ac.uk

Supplement materials

Supplementary Discussion 1
Trajectory calculation of the laser pulse's centroid motion
In our considered situation, the plasma density distribution only depends on the radical coordinate. According to the eikonal equation and laser group velocity equation $v_g = c \cdot n(r)$, in cylindrical coordinates $(r,\ \phi,\ z)$, we have

$$\frac{d\vec{R}}{dt} = \frac{c^2}{\omega_0}\vec{k} \tag{S1}$$

$$\frac{d\vec{k}}{dt} = -\frac{\omega_0}{2}\frac{\partial}{\partial\vec{R}}\left(\frac{n(r)}{n_c}\right) \tag{S2}$$

where ω_0 and $\vec{k}$ are the frequency and wavenumber vector of the laser pulse respectively, n_c is the critical density of plasma. Rewriting Eq. (S1) and

Eq. (S2) in cylinder geometry, we get

$$\frac{dr}{dt} = \frac{c^2}{\omega_0} k_r \tag{S3}$$

$$r\frac{d\phi}{dt} = \frac{c^2}{\omega_0} k_\phi \tag{S4}$$

$$\frac{d}{dt} \ln (k_\phi \cdot r) = 0 \tag{S5}$$

$$\frac{dk_z}{dt} = 0 \tag{S6}$$

Based on Eq. (S5) and Eq. (S6), $k_\phi r$ and k_z are constant since the laser pulse enters a channel

$$k_\phi = k_0 \cdot (1 - n_{e0}/n_c)^(1/2) \cdot sin\theta_z \cdot b \tag{S7}$$

$$k_z = k_0 \cdot (1 - n_{e0}/n_c)^(1/2) \cdot cos\theta_z \tag{S8}$$

where $n_{e0} = n_e(r = \sqrt{x_0^2 + y_0^2})$ is the plasma density at the point of laser entrance with x_0 and y_0 are laser initial off-axis distances,
$\theta_z = arccos(\sqrt{1 - cos^2\theta_x - cos^2\theta_y})$ with $\theta_{x,\ y,\ z}$ the angles between laser propagation direction and the space coordinate axes x, y, and z, and $b = (y_0 - x_0 cos\theta_y / cos\theta_x)/(\sqrt{(cos\theta_y/cos\theta_x)^2 + 1})$ is called the "striking distance", which is defined as the distance between the projection of the incident laser pulse and the point of the plasma channel centre on the incident surface. Substitute $k_r = \sqrt{k_0 \cdot (1 - n_{e0}/n_c)^{1/2} - k_\phi^2 - k_z^2}$ and k_ϕ into Eq. (S3) and Eq. (S4), one can obtain the trajectory equation

$$\frac{dr}{dz} = \pm\frac{1}{cos\theta_z}[1 - \frac{n(r)}{n_c} - (1 - \frac{n_{e0}}{n_c}) \cdot (sin^2\theta_z \cdot \frac{b^2}{r^2} + cos^2\theta_z)]^{1/2} \tag{S9}$$

$$\frac{d\phi}{dz} = \frac{bsin\theta_z}{r^2 cos\theta_z} \tag{S10}$$

In Eq. (S9), when the expression inside the square brackets equals zero, the electrons are at the points with the largest radial coordinate r and the choice of plus-minus sign should be changed.

Supplementary Discussion 2
Simulation of ionization injected electrons and their radiation from a helical undulator

To justify the feasibility of our scheme adaptable to the self-injected electrons, a non-optimized simulation by using ionization injection mechanism has been carried out. In this simulation, the duration of the laser pulse is $L_0 = 6.0\ T_0$ and the incidence parameters are $x_0 = 1\ \mu m$, $y_0 = 0$, $\theta_x = 89°$ and $\theta_y = 91°$. The on-axis density of the plasma channel is $n_0 = 0.004\ n_c$. The density of the partially ionized nitrogen is $n_{N^{5+}} = 8.0 \times 10^{-4}\ n_c$ which

is used for injection purpose. The nitrogen gas is located from $x = 30\ \lambda_0$ to $x = 60\ \lambda_0$ with an up-ramp plateau down-ramp ($5\ \lambda_0 - 20\ \lambda_0 - 5\ \lambda_0$) profile.

In the simulation, we have found that finally about 2 pC electrons are ionization injected and accelerated to about 160 MeV. Typical trajectories of the injected electrons are shown in Fig. S1 and the distribution of the electrons radiation and polarization are plotted in Fig. S2. They are basically similar to the external injected electrons radiation. It should be mentioned that due to the extreme numerical simulation cost for ionization injection in our 3D simulation case, we have not optimized the incidence parameters. However, the current simulation basically justifies the reasonability of using the typical external injection beam in the paper.

Supplementary Discussion 3
Relativistic non-linear effects analysis
To see relativistic non-linear effects, we have made a few more simulations by using small a0. Fig. S3(a) shows the comparison of laser centroid trajectories in theory (black lines), $a_0 = 2$ simulation (red lines), and $a_0 = 0.5$ simulation (green lines). It is clear that without the non-linear effect the $a_0 = 0.5$ simulation trajectory perfectly matches the theoretical trajectory. Whereas in $a_0 = 2$ situation, simulation trajectory deviates from that of theory as the laser pulse propagating mainly in oscillation period. Fig. S3(b) shows the comparison of electron beam centroid trajectories in $a_0 = 2$ simulation (blue lines) and $a_0 = 0.5$ simulation (magenta lines). The electron beam motion in the bubble shaped wakefield has to follow the oscillation process of the laser pulse. So the oscillation amplitude becomes smaller and the oscillation period becomes more constant in the $a_0 = 2$ simulation. In summary, the relativistic non-linear effect indeed exists in our simulations. It leads to the laser centroid trajectory and the electron beam centroid trajectory deviate to a certain extent from that in theory and in linear regime. However, the general trend remains unchanged and the helical motion of such trajectories are still stable under this parameter.
Supplementary Discussion 4
Further Radiation spectra analysis
The energy spectrum for the point with lowest intensity, which is on the elliptical ring with the azimuth angle ϕ of 45° (see Fig. 3(a)), is shown in Fig. S4(a). The peak energy at this point is smaller than that of the points with maximum radiation intensities. This is consistent with the peak energy equation $E_p = 3\pi\gamma^3 r_{os}hc/\Lambda_{os}$ when γ and r_{os} are smaller. And Fig. S4(b) shows the energy spectrum integrated over the entire observe region. This spectrum is wider than the radiation spectrum of the marked points in the manuscript (see Fig. 3(c) and (d)). Because the electrons are being accelerated during the helical motion. Electrons with different energy would radiate photons with different energy as well. And the radiation intensity

in x direction and y direction are almost the same which is also reasonable to the total integral area.

Supplementary Movie 1
The spiral motion of the laser pulse, the bubble structure and accelerated electrons in 3D-PIC simulations

Figures

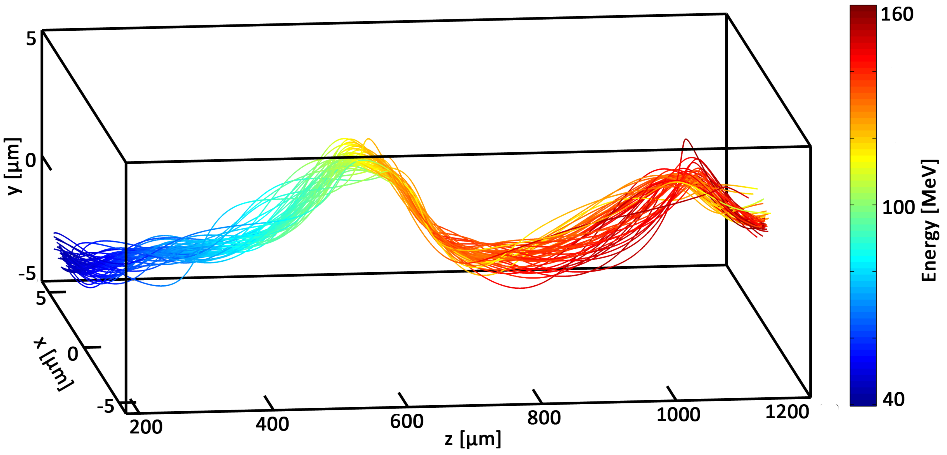

Fig. S1 **Spiral trajectories of the injected and accelerated electrons from the ionization injection mechanism in the plasma channel.**

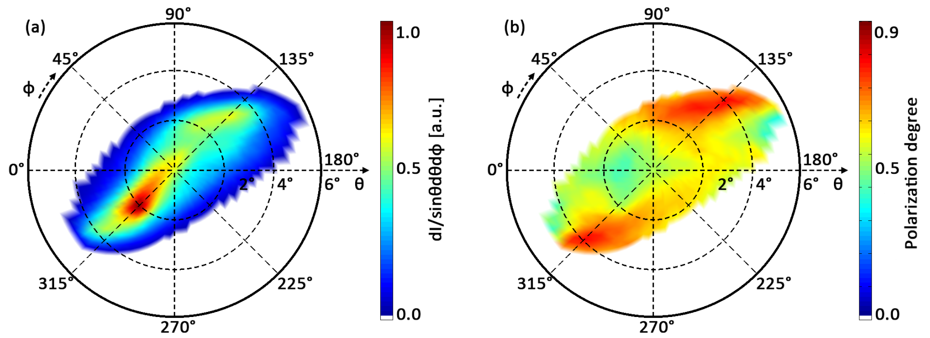

Fig. S2 **Distribution of intensity (a) and polarization (b) of the far field radiation.**

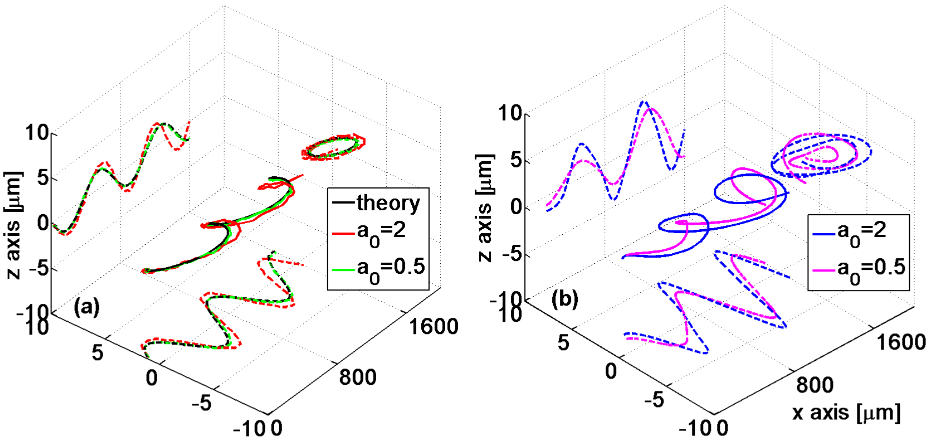

Fig. S3 **(a) Comparison of laser centroid trajectories in theory (black lines), $a_0 = 2$ simulation (red lines), and $a_0 = 0.5$ simulation (green lines). (b) Comparison of electron beam centroid trajectories in $a_0 = 2$ simulation (blue lines) and $a_0 = 0.5$ simulation (magenta lines)**

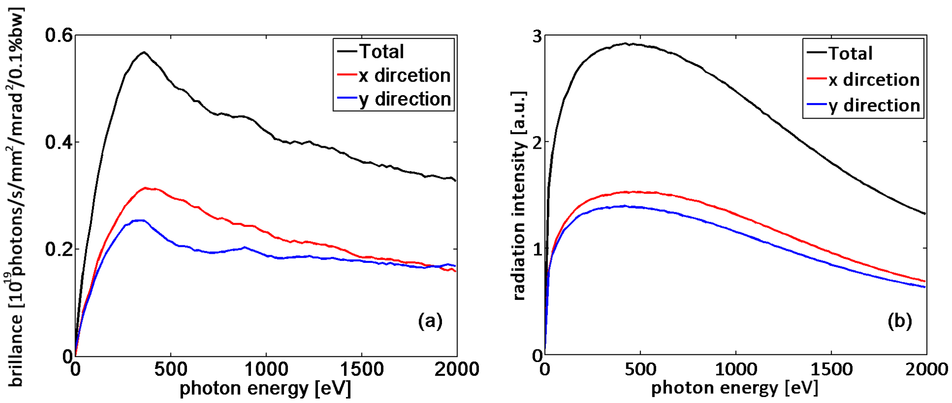

Fig. S4 **Energy spectrum for the point with lowest intensity (a) and integrated over the entire observation region (b).**
